# Cofilin Inhibitor Improves Neurological and Cognitive Functions after Intracerebral Hemorrhage by Suppressing Endoplasmic Reticulum Stress Related-Neuroinflammation

**DOI:** 10.3390/ph17010114

**Published:** 2024-01-15

**Authors:** Daniyah A. Almarghalani, Ghaith A. Bahader, Mohammad Ali, L. M. Viranga Tillekeratne, Zahoor A. Shah

**Affiliations:** 1 Department of Pharmacology and Experimental Therapeutics, College of Pharmacy and Pharmaceutical Sciences, The University of Toledo, Toledo, OH 43614, USA; 2 Department of Medicinal and Biological Chemistry, College of Pharmacy and Pharmaceutical Sciences, The University of Toledo, Toledo, OH 43614, USA

**Keywords:** cofilin inhibitor, ICH, neuroinflammation, microglial activation, post-stroke cognitive impairment, cofilin rods/aggregates, inflammasome, endoplasmic reticulum

## Abstract

Neuroinflammation after intracerebral hemorrhage (ICH) is a crucial factor that determines the extent of the injury. Cofilin is a cytoskeleton-associated protein that drives neuroinflammation and microglia activation. A novel cofilin inhibitor (CI) synthesized and developed in our lab has turned out to be a potential therapeutic agent for targeting cofilin-mediated neuroinflammation in an in vitro model of ICH and traumatic brain injury. The current study aims to examine the therapeutic potential of CI in a mouse collagenase model of ICH and examine the neurobehavioral outcomes and its mechanism of action. Male mice were subjected to intrastriatal collagenase injection to induce ICH, and sham mice received needle insertion. Various concentrations (25, 50, and 100 mg/kg) of CI were administered to different cohorts of the animals as a single intravenous injection 3 h following ICH and intraperitoneally every 12 h for 3 days. The animals were tested for neurobehavioral parameters for up to 7 days and sacrificed to collect brains for hematoma volume measurement, Western blotting, and immunohistochemistry. Blood was collected for cofilin, TNF-α, and IL-1β assessments. The results indicated that 50 mg/kg CI improved neurological outcomes, reversed post-stroke cognitive impairment, accelerated hematoma resolution, mitigated cofilin rods/aggregates, and reduced microglial and astrocyte activation in mice with ICH. Microglia morphological analysis demonstrated that CI restored the homeostasis ramification pattern of microglia in mice treated with CI. CI suppressed endoplasmic reticulum stress-related neuroinflammation by inhibiting inflammasomes and cell death signaling pathways. We also showed that CI prevented synaptic loss by reviving the pre- and post-synaptic markers. Our results unveil a novel therapeutic approach to treating ICH and open a window for using CI in clinical practice.

## 1. Introduction

Intracerebral hemorrhage (ICH), a subtype of hemorrhagic stroke, accounts for 15% of all strokes that result from the rupture of cerebral vessels and accumulation of blood within the brain parenchyma [1]. ICH significantly contributes to stroke-related morbidity, mortality, neurologic disability [2], and cognitive decline [3,4]. After ICH, only half of the patients survive up to 1 year, and patients who survive suffer from neurologic deficits that negatively impact their lives [5]. The pathophysiology of ICH-elicited injury encompasses primary and secondary brain injury [6]. Primary injury involves mechanical pressure and mass effects followed by hematoma expansion, edema, and swelling, causing brain tissue disruption [6]. The secondary injury further exaggerates the prevailing pathological conditions and is considered a devastating stage of ICH, depending on the location of the ictus, and determines the rate of recovery [7,8]. Complex pathological cascades are responsible for secondary injury, including erythrocyte lyse releasing of hemoglobin, iron, and heme, generating free radicals like reactive oxygen species (ROS), and increased cytotoxicity. An overwhelming accumulation of ROS interrupts endoplasmic reticulum (ER) homeostasis, triggering apoptosis and activating neuroinflammatory signaling cascades by the interaction of unfolded protein response components and cytokine transcription factors [9]. In addition, ER stress activates the NOD-like receptor of the pyrin domain containing 3 (NLRP3) inflammasome in a complement-induced neuroinflammation manner following ICH by releasing interleukin-1β (IL-1β) and interleukin-18 (IL-18) as well as overexpressing caspase-1 [10]. Subsequently, the activated resident microglia and formed classic M1-like phenotype (proinflammatory) secrete a wide range of proinflammatory cytokines, such as IL-1β, interleukins-6 (IL-6), and tumor necrosis factor (TNF-α), which ultimately prime neuroinflammation, leading to the infiltration of leukocytes/neutrophils/macrophages from the peripheral circulation to the ictus side and further deteriorating the ICH-affected brain [11]. Excessive neuroinflammatory response potentially amplifies brain injury by neuronal apoptosis, interruption of the blood–brain barrier (BBB), microglia activation, brain edema, and axial damage [10,12]. Notably, the protracted features of neuroinflammation following ICH offer a window of opportunity for developing therapies to mitigate its detrimental consequences. To date, surgical evacuation of hematoma and supportive treatment is the only therapeutic approach available, and there is a lack of targeted therapeutic interventions specifically for the treatment of ICH [1]. Prevention of hematoma expansion and reduced neuroinflammation are the potential therapeutic targets against ICH [12,13].

Cofilin is an actin-associated depolymerizing protein that regulates actin dynamics [14]. Several kinases and phosphatases control cofilin phosphorylation and dephosphorylation. LIM and TES kinases (LIMK and TESK) activate cofilin by phosphorylation at serine-3 residue (Ser3). However, slingshots (SSH), phosphoprotein phosphatases (PP1/PP2A), and chronophin reactivate cofilin [15,16,17,18]. The persistent activation of cofilin leads to saturated actin filaments and provokes the formation of cofilin rods/aggregates, which triggers neurotoxicity, neuroinflammation, microglia activation, damage synapses, and initiates dendritic spine loss, prompting post-stroke cognitive impairment (PSCI) [19,20,21,22]. Cofilin rods/aggregates result from an increase in the ratio of cofilin/F-actin, the dephosphorylation of cofilin, oxidative stress, microglia activation, and tau pathology [21,23,24]. Cofilin overexpression facilitates the cofilin translocation to mitochondria and induces mitochondrial permeability, which releases cytochrome C and triggers caspases [25,26]. Our lab showed that the knockdown of cofilin increased cell survival in neurons [27] and attenuated microglia activation [27,28]. In addition, we demonstrated that the blockade of cofilin by siRNA in a mouse collagenase model of ICH diminished neuronal death, oxidative stress, and microglia activation and improved neurobehavioral dysfunction [29]. Our synthesized novel cofilin inhibitor (CI) significantly increased neuronal survival by decreasing caspase-3, nuclear factor-κB (NF-κB), and high-temperature requirement and remarkably prevented microglial activation and neuroinflammation by reducing nitric oxide and TNF-α in an in vitro ICH model [30]. Our recent study showed that cofilin was overexpressed and colocalized within microglia around the hemorrhagic zone in human autopsy brain sections, and the longitudinal mouse study up to 28 days of ICH demonstrated that cofilin was upregulated on days 1 and 3 around the hematoma and on day 7, cofilin dysfunction paved the way for rods/aggregates [20].

Therefore, this study explored the role and mechanism of CI following ICH in mice. We assessed motor and PSCI after treatment with different doses of CI (25, 50, and 100 mg/kg) in different cohorts of mice. Finally, we investigated the 50 mg/kg dose of CI and its impact on mitigating neuroinflammation and other signaling parameters. Our data provide a solid justification for targeting cofilin as a novel therapeutic target for ICH.

## 2. Results

1. Cofilin inhibitor treatment enhances neurobehavioral recovery after ICH in mice.

**Figure 1:**
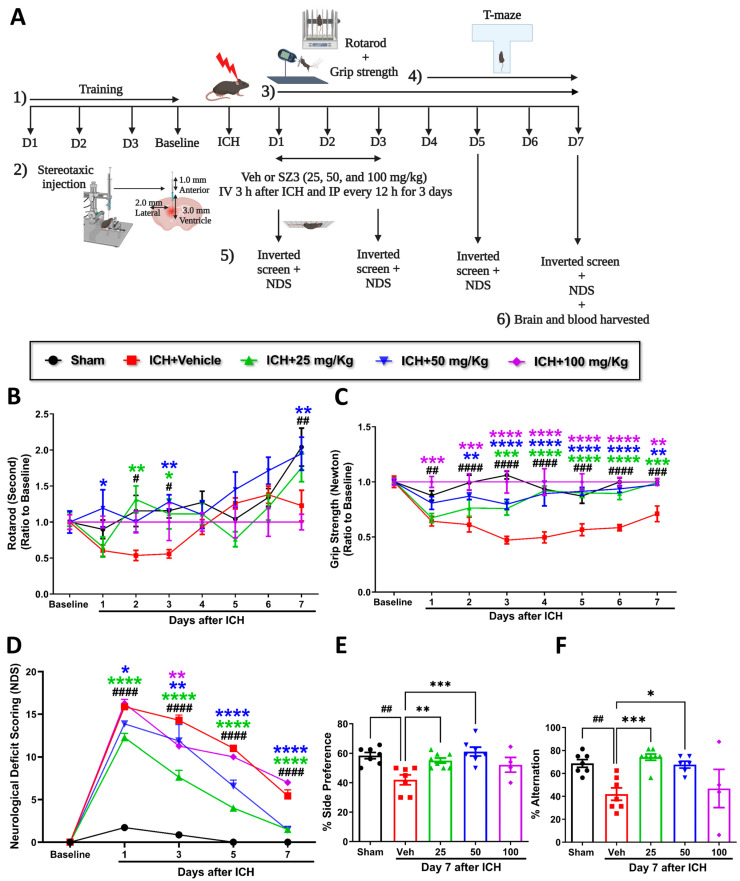
Cofilin inhibitor improved neurobehavioral functions. Effects of different doses of CI on neurobehavioral recovery after ICH in mice. (**A**) The flow chart shows the experimental design. (**1**) Mice were trained on the rotarod, grip strength, and inverted screen 3 days and 24 h before the surgery (baseline, each animal was assessed thrice per trial). (**2**) Different groups of mice were subjected to intrastriatal collagenase injection-induced ICH and then received different doses of CI (25 mg/kg, 50 mg/kg, and 100 mg/kg). Mice received intravenous (i.v.) injections 3 h. after ICH (25, 50, or 100 mg/kg) or an equivalent amount of the vehicle followed by intraperitoneal (i.p) injections every 12 h. for 3 consecutive days. Sham mice had an insertion of only the needle (a total of 50 mice were used in the study). (**3**) Rotarod and grip strength were scored for all animals and compared among ICH + vehicle and ICH + CI (25, 50, or 100 mg/kg) groups 24 h before the ICH (baseline) and from day 1 to day 7. (**4**) PSCI was determined by T-maze from day 4 to 7). (**5**) Inverted screen and NDS were evaluated for all mice and compared among ICH + vehicle and ICH with different doses of CI groups before 24 h and at days 1, 3, 5, and 7 after ICH. (**6**) Mice were sacrificed, and brains and blood samples were collected on day 7. (**B**) Rotarod latency time showed significant improvement on days 2 and 3 in CI 25 mg/kg and on days 1, 3, and 7 in CI 50 mg/kg compared to the vehicle. (**C**) Forelimb strength markedly enhanced in CI doses of 25, 50, or 100 mg/kg. (**D**) NDS showed improvement with 25 and 50 mg/kg of CI. (**E**,**F**) Summary data for the performance of sham and ICH + vehicle and ICH + CI (25, 50, or 100 mg/kg) groups on side preference and alternation in the T-maze task. Mice treated with CI (25 mg/kg and 50 mg/kg) significantly improved spatial learning and memory (increased % of side preference and % of alternation). No significant improvement was noted in the 100 mg/kg treated mice. (* Difference within CI groups relative to vehicle group (* *p* < 0.05, ** *p* < 0.01, *** *p* < 0.001, **** *p* < 0.0001). ^#^ Difference in sham and vehicle groups after ICH (^#^ *p* <0.05, ^##^ *p* < 0.01, ^###^ *p* < 0.001, ^####^ *p* < 0.0001, (n = 7–6 mice per group). Two-way ANOVA followed by Sidak’s *post hoc* comparisons).

Different concentrations of CI (25, 50, and 100 mg/kg)-treated mice were assessed for rotarod, grip strength, inverted screen, and neurological deficits at baseline and at different time points after ICH. T-maze was performed from day 4 to day 7 to evaluate spatial learning and memory (Figure 1A shows the experimental design). Baseline performance on the rotarod, grip strength, and the inverted screen were not altered among the mice groups. Mice treated with CI showed significant improvement in rotarod performance on days 2 and 3 with 25 mg/kg and days 1, 3, and 7 with 50 mg/kg compared with the vehicle. The CI group with 100 mg/kg showed no differences compared to the vehicle group (Figure 1B).

Furthermore, CI treatment enhanced limb grip strength in 25 mg/kg, 50 mg/kg, and 100 mg/kg groups compared to vehicle (Figure 1C). CI treatment at a concentration of 50 mg/kg significantly increased the time on the inverted screen on days 1 and 3 relative to the vehicle (Figure 1A and Figure S1). Mice in the vehicle-treated group suffered from neurologic deficits, whereas 25 mg/kg and 50 mg/kg treatment significantly reversed the deficits on days 1, 3, 5, and 7. No significant improvement was observed in the 100 mg/kg treatment group compared to the vehicle (Figure 1D). There was 60% mortality observed with a higher concentration of CI (100 mg/kg) (Figure S2). These findings show that CI markedly rescued neurobehavioral deficits in ICH mice. We did not observe the effect of CI on body weight compared to the vehicle group after ICH (Figure 1B and Figure S1).

The cofilin level in the plasma and TNF-α and IL-1β levels in the serum were decreased in the different concentrations of CI relative to the vehicle group; however, the data were not statistically significant (Figure 1C–E and Figure S1). Cognitively, mice subjected to ICH (vehicle) showed a substantial decrease in the percent of alternation and the percent of side preference compared to the sham group. Compared to the vehicle, treatment with CI (25 mg/kg and 50 mg/kg) significantly enhanced spatial learning and memory. No significant improvement was observed in the 100 mg/kg treated mice (Figure 1E,F).

2. Cofilin inhibitor treatment improves hematoma resolution after ICH in mice.

**Figure 2:**
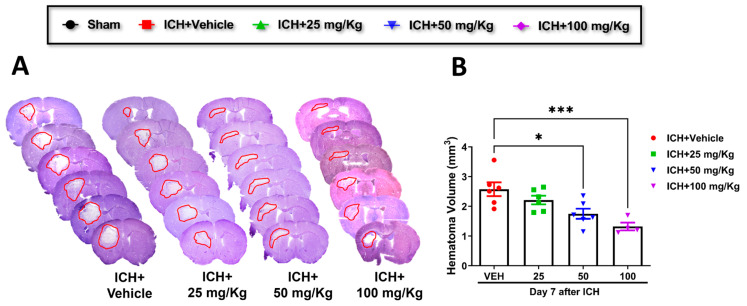
Cofilin inhibitor reduced hematoma volume. (**A**,**B**) Quantification of hematoma lesion volume, visible in red regions in mice treated with CI (25, 50, or 100 mg/kg) or vehicle at day 7 after ICH. (* Difference within CI groups relative to vehicle group (* *p* < 0.05, *** *p* < 0.001). (n = 7–6 mice per group). Two-way ANOVA followed by Sidak’s *post hoc* comparisons).

Consistent with improving motor and cognitive performance, CI significantly reduced hematoma volume in 50 mg/kg and 100 mg/kg groups relative to the vehicle group. A reduction trend was observed in 25 mg/kg mice compared to the vehicle, although the data were insignificant (Figure 2A,B). Based on these results, we selected CI 50 mg/kg dose to further investigate the in-depth molecular mechanism.

3. Cofilin inhibitor reduces cofilin rods/aggregates, perihematomal glial activation, and oxidative/nitrosative stress after ICH.

**Figure 3:**
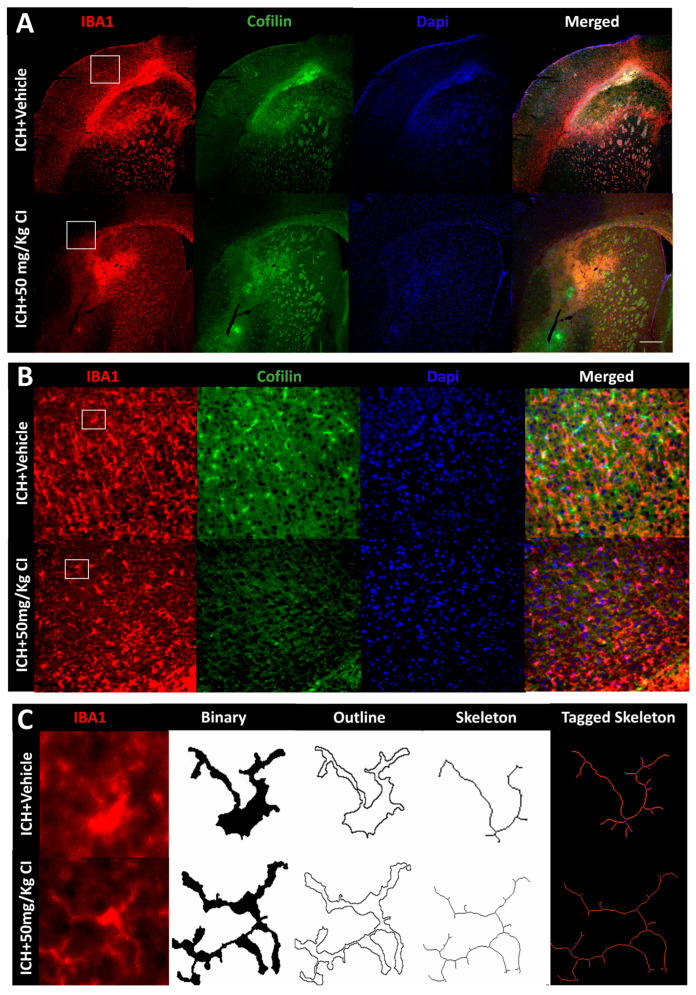

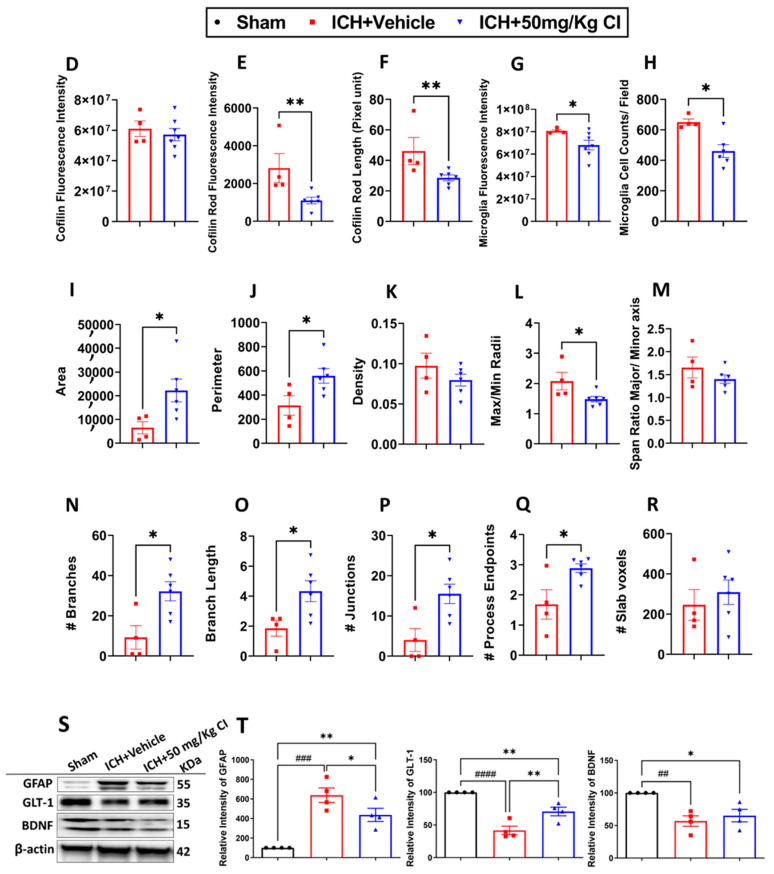
Cofilin inhibitor reduced cofilin rods/aggregates, microgliosis, and astrocytosis. (**A**–**C**) Impact of CI (50 mg/kg) on cofilin rods/aggregates, microglial activation, and microglia morphology. Anti-cofilin (a marker of active cofilin) and anti-IBA1 (a marker of active microglia/macrophage) were used. Immunohistochemistry analysis showed that CI mitigated the cofilin rods/aggregates (**D**–**F**) and microglial activation (**G**,**H**) around hemorrhagic areas compared to the vehicle. (**C**) White squares in A represent the focus area for B; white square in B represent the focus area for C. Pictures in subfigure A was illustrated at 4x magnification, subfigure (**B**) at 20× magnification, and subfigure (**C**) at 100× magnification. Analysis of microglia morphology: binary images were outlined and then skeletonized to examine the morphological alteration of microglia in the ICH + vehicle and ICH +CI groups using ImageJ Frac Lac plugin protocols. (**I**–**R**) Statistical analysis of microglial morphology (shape, complexity, number of branches, and process) using FracLac. (**S**,**T**) β-actin was used as loading control; GFAP, GLT-1, and BDNF were used as markers of activated astrocytes, astrocytic glutamate transporter-1, and brain-derived neurotrophic factors. (**T**) GFAP protein levels were decreased, while GLT-1 was markedly increased in the CI-treated group. No significant variation was observed in the BDNF among groups. Data are indicated as mean ± SEM, where *p* < 0.05 was considered significant (n = 4–6/group, unpaired Student’s *t*-test or one-way ANOVA followed by Sidak’s *post hoc* comparisons). * Difference in ICH + CI group relative to ICH + vehicle group (* *p* < 0.05, ** *p* < 0.01) and ^#^ Difference in ICH + vehicle compared to the sham group (^##^ *p* < 0.01, ^###^ *p* < 0.001, ^####^ *p* < 0.0001).

To explore the impact of CI on cofilin rods/aggregates, microgliosis, astrogliosis, and oxidative/nitrosative stress on day 7, we performed immunohistochemistry and Western blotting (WB) on brain tissues of the ipsilateral region. Compared to the vehicle, the measurement of cofilin fluorescence intensity decreased by CI treatment; nonetheless, the data was insignificant (Figure 3A,D). The cofilin rod intensity and cofilin rod length were markedly reduced with CI treatment (Figure 3B,E,F). The immunofluorescence intensity and the counts of IBA1-positive cells were significantly reduced with CI treatment compared to the vehicle (Figure 3A,B,G,H). To investigate the effect of CI on morphological consequences of microglial activation after ICH in detail, we measured microglia area, perimeter, span ratio, radius length, and branches using ImageJ. Treatment with CI increased the area and perimeter and reduced the thickness, span ratio, and max/min radius length (Figure 3C,I–M). Microglial branches, junctions, process endpoints, slab voxels, and length were increased in the CI-treated group relative to the vehicle, suggesting that CI restored microglia morphology to the ramified stage (Figure 3C,N–R).

Noting the decrease in astrocytes in the CI-treated group compared to the vehicle (Figure 3S,T), we further tested the expression of astrocytic glutamate transporter 1 (GLT-1) and brain-derived neurotrophic factor (BDNF), part of neuroprotective machinery. Interestingly, GLT-1 significantly recovered after treatment with CI; however, no remarkable alteration was detected in the expression of BDNF (Figure 3S,T). To investigate other markers of injury, such as oxidative/nitrosative stress-inducible nitric oxide synthase (iNOS), we observed that mice treated with CI had lower 3-nitrotyrosine (3-NT) co-localization with iNOS around the hemorrhagic area compared to the vehicle (Figure S3). This suggests that CI attenuated the accumulation of cofilin rods/aggregates, microglia, astrocytes, and oxidative/nitrosative stress after ICH.

4. Effect of Cofilin inhibitor treatment on cofilin signaling after ICH.

**Figure 4:**
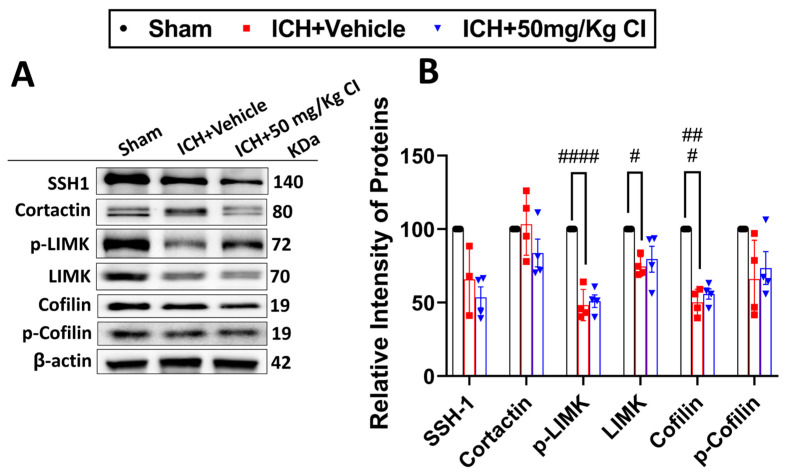
Effect of cofilin inhibitor on cofilin signaling. (**A**) β-actin was used as loading control; SSH1, cortactin, p-LIMK, LIMK, cofilin, and p-cofilin were used as markers for cofilin signaling. (**B**) Illustrative WB analysis of cofilin signaling after ICH in the ipsilateral region showed that cofilin signaling was not significantly altered with CI treatment compared to the vehicle group. (One-way ANOVA followed by Sidak’s *post hoc* comparisons). Data are displayed as mean ± SEM, where *p* < 0.05 was considered significant. # Difference in ICH + vehicle compared to the sham group (^#^ *p* < 0.05, ^##^ *p* < 0.001, ^####^ *p* < 0.0001). (normalized to B-actin, n = 4 mice per group).

We explored upstream and downstream cofilin signaling after CI treatment. No significant differences were detected in the cofilin signaling, including SSH1, cortactin, p-LIMK, LIMK, cofilin, and p-cofilin in the CI-treated mice compared to vehicle mice on day 7 (Figure 4A,B).

5. Cofilin inhibitor treatment reduces ER stress-related neuroinflammation by preventing NLRP3 inflammasome and reducing cell death after ICH.

**Figure 5:**
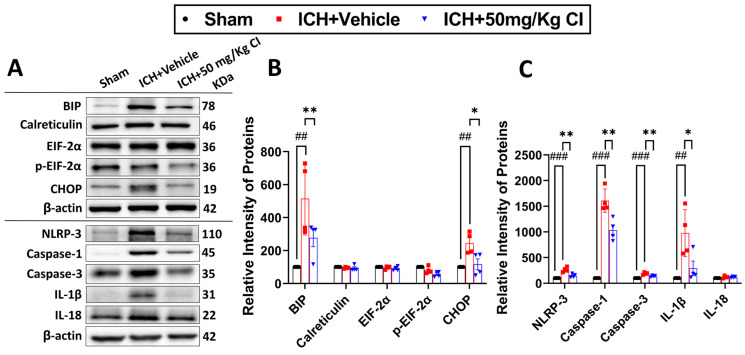
Cofilin inhibitor reduced ER stress-related neuroinflammation by inhibiting NLRP3 inflammasome pathways and cell death. (**A**) β-actin was used as loading control; BIP, calreticulin, EIF-2α, p-EIF-2α, and CHOP were used as markers for the ER stress, NLRP-3, caspase-1, IL-1β, and IL-18 were used as markers for inflammasome, caspase-3 was used as an apoptotic marker (normalized to β-actin, n = 4 mice per group). (**B**) WB analysis of ER stress, including BIP and CHOP, was markedly reduced in the CI-treated group. No significant difference was observed in the calreticulin, EIF-2α, and p-EIF-2α. (**C**) NLRP-3, caspase-1, and IL-1β were significantly decreased in the CI-treated group; however, IL-18 was not changed. Caspase-3 was also notably decreased in CI treatment. (One-way ANOVA followed by Sidak’s *post hoc* comparisons). Data are indicated as mean ± SEM, where *p* < 0.05 was considered significant. * Difference in ICH + CI group relative to ICH + vehicle group (* *p* < 0.05, ** *p* < 0.01) and ^#^ Difference in ICH + vehicle compared to the sham group (^##^ *p* < 0.01, ^###^ *p* < 0.001).

To ascertain the potential downstream effects of cofilin inhibition, we investigated neuroinflammation mediated by NLRP3 inflammasome activation. Binding immunoglobulin protein (BIP) and C/EBP homologous protein (CHOP) of the ER stress pathway were upregulated in the vehicle, whereas CI treatment significantly reversed the increase in these two proteins. The expression of calreticulin, eukaryotic initiation factor 2 alpha kinases (EIF-2α), and p-EIF-2α did not show significant changes in all groups (Figure 5A,B).

On day 7 following ICH, we found that CI treatment decreased the protein expression of NLRP3, caspase-1, and IL-1β. Interestingly, CI does not affect IL-18 after ICH (Figure 5A,C). To test the effect of CI treatment on apoptosis, caspase-3 expression was found to be increased in the vehicle; however, CI treatment significantly reduced caspase-3 levels (Figure 5A,C).

6. Cofilin inhibitor treatment rescues pre-/post-synaptic loss after ICH.

**Figure 6:**
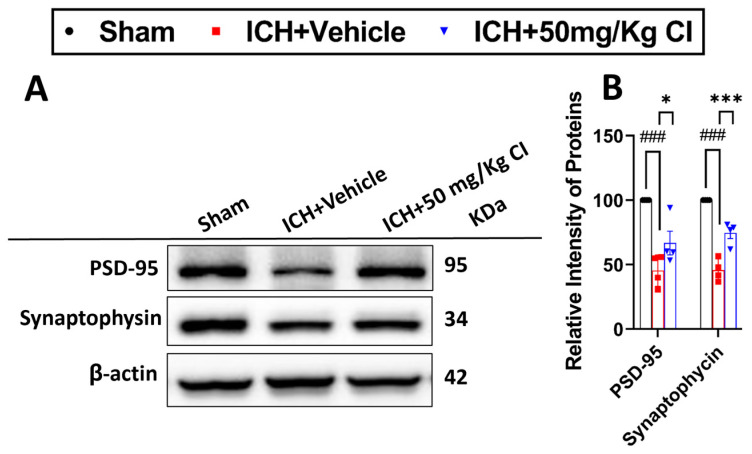
Cofilin inhibitor prevented synaptic loss. (**A**) β-actin was used as loading control; PSD95 and synaptophysin were used as post-synaptic and presynaptic markers, respectively. (**B**) WB analysis of PSD-95 and synaptophysin was increased in the CI-treated group. Data are shown as mean ± SEM, where *p* < 0.05 was considered significant. * Difference in ICH + CI group relative to ICH + vehicle group * *p* < 0.05, *** *p* < 0.001) and ^#^ Difference in ICH + vehicle compared to the sham group (^###^ *p* < 0.001). (normalized to B-actin, n = 4 mice per group).

Based on our earlier results that show an improvement in cognitive function in the T-maze test, we assessed the effect of CI on synapse density in ICH mice by evaluating the levels of two synaptic markers, post-synaptic density protein-95 (PSD-95) and synaptophysin. Our results showed that CI rescued synapse loss by significantly increasing the expression of both proteins in the CI-treated group compared to the vehicle (Figure 6A,B).

## 3. Discussion

In the present study, we investigated the therapeutic effect of CI after ICH. We present evidence that a novel CI attenuates ICH brain injury via different pathways. Our results indicated that 50 mg/kg CI improved neurological outcomes, reversed post-stroke cognitive impairment, accelerated hematoma resolution, mitigated cofilin rods/aggregates, and reduced microglial and astrocyte activation in mice following ICH. Our results also demonstrated that CI suppressed endoplasmic reticulum stress-related neuroinflammation by inhibiting inflammasomes and cell death signaling pathways. Finally, CI was observed to prevent synaptic loss by reviving the pre- and post-synaptic markers. The study’s findings suggest that cofilin plays a crucial role in microglial activation, neuroinflammation, and cognition in ICH; its targeting with CI has potential therapeutic value in decreasing the impact of ICH injury.

The mechanism of ICH injury is intricate, encompassing diverse signaling pathways and involving various cell types [31]. In our previously published studies, we have highlighted the role of cofilin in mediating ICH pathophysiology [20,29]. In our recent study that explored the alternation of cofilin expression following ICH in mice, we determined that cofilin is overexpressed from day 1 to 3, and rods start accumulating on day 7 after ICH [20]; therefore, we selected CI treatment for 3 days only and 7 days survival to analyze the therapeutic potential of CI [20] and study its mechanism of action. Neurobehavioral deficits are major comorbidities associated with ICH [32]. Here, we found a significant reduction in hematoma volume following the administration of increasing concentration of CI, which underscores the effectiveness of the early inhibition of cofilin during the acute phase following ICH. This treatment option translated into a significant improvement in motor, sensory, and other neurobehavioral functions, which is in consonance with our previous published reports [29].

It is evident that the alternation of cofilin/p-cofilin dynamics and its upstream signaling cascades, such as SSH1 and LIMK, is involved in the pathophysiology of different neurodegenerative diseases [33,34,35]. Likewise, dysregulation of other actin-related proteins, such as cortactin, has been shown to play an essential role in mediating ischemic stroke injury by preserving mitochondrial stability and survival in astrocyte cells [36]. Results from this study showed that only cofilin and LIMK/p-LIMK had reduced 7 days after ICH with no significant differences in the CI-treated group. This implies the selectivity of CI on cofilin and its upstream pathway and is consistent with our previous study [20].

Neuroinflammation, oxidative/nitrosative stress, and glial cell activation are fundamental mechanisms involved in the ICH pathophysiology [37]. A common pathway has been discovered in various neurodegenerative diseases, such as Alzheimer’s disease [38], Parkinson’s disease [39], Huntington’s disease [40], traumatic brain injury (TBI) [41], ischemic stroke, and ICH [20]. This pathway involves the dysregulation of cofilin signaling or the formation of rod-shaped inclusions, known as rods/aggregates, in the dendrites, axons of neuronal cells, and microglia [20,42]. The rods/aggregates are formed when the actin-severing protein, cofilin, binds to actin, forming 1:1 bundles [34,43]. Preclinical studies in ischemic stroke rodent models showed that the formation of cofilin–actin rods is implicated in the pathophysiology of the disease via oxidative stress mechanisms, and the inhibition of rods formation is associated with reduced apoptosis following ischemic stroke [44,45]. Moreover, we recently demonstrated that cofilin rods/aggregate formation in human ICH autopsy and mice brain sections are associated with microglial morphology alterations and neuroinflammatory responses following ICH [20]. In addition, we explored the impact of CI in oxidative stress conditions, including in vitro and in vivo studies. Our findings indicate that exposure to H_2_O_2_ leads to an upregulation of cofilin and SSH1, an upstream regulator of cofilin signaling in microglia cells; however, CI significantly reduces the expression of cofilin and SSH1, subsequently mitigating the microglial activation by diminishing the release of inflammatory mediators such as TNF-α, NF-κB, and high mobility group box 1 and ROS level. In a mouse model of TBI, CI treatment notably activates nuclear factor erythroid 2-related factor 2 and reduced the expression of oxidative/nitrosative stress markers at both the protein and gene levels, suggesting that CI has a protective effect against oxidative and nitrosative stress in TBI [41]. These findings highlight the significance of inhibiting cofilin and rod formation as an option for overcoming microglial activation and neuroinflammation. In this context, we demonstrated that CI effectively reduced cofilin rod/aggregates and restored the microglial morphology to the ramified resting stage. Additionally, CI reduced astrocyte activation and neuronal excitotoxicity, emphasizing the role of cofilin overexpression and rods/aggregate formation in promoting neuroinflammatory pathological responses following ICH.

Another important pathological mechanism is the activation of an ER-related unfolded protein response (UPR) quality control mechanism due to the accumulation of unfolded and misfolded proteins in response to ICH injury [9]. ICH activates the ER stress pathway, leading to neurological impairments via crosstalk with cell death [9]. Pyroptosis, a recently discovered form of cell death, has received consideration because of its impact on various diseases, like ischemic stroke [46], hemorrhagic stroke [47], and TBI [48]. Inhibition of ER stress by tauroursodeoxycholic acid provided neuroprotective effects and reduced neuronal pyroptosis by diminishing the expression of interleukin-13 after ICH [49]. Ren et al. have shown that NLRP3 is activated after ICH and promotes brain edema and neuroinflammation. A potent NLRP3 compound, MCC950, inhibits NLER3 activation and attenuates inflammation and brain injury after ICH [50]. Interestingly, the current study found that CI partially alleviated ICH pathology by reducing ER stress markers and pyroptosis cell death. It is worth mentioning that an investigation has reported the cofilin–actin depolymerization factor as one of the diverse protein classes that are secreted following toll-like receptor (TLR4) and NLRP3 activation in macrophages [51]. Moreover, another recent study has shown that cofilin mediates acute kidney injury by promoting the ER stress pathway in an oxygen–glucose deprivation cell model [52]. These studies are in agreement with the results reported in the current study about the role of cofilin in aggravating ICH injury via these distinct pathways.

PSCI is a major comorbidity frequently occurring in the acute and chronic phases following ICH [53]. Multiple potential mechanisms have been suggested behind the PSCI, including reduced dendritic spine density, impaired synaptic plasticity [54], neuroinflammation, glial cell activation [55], and neuronal excitotoxicity [56]. Impaired neuronal synaptic structure and function were linked to cofilin rod formation in an in vitro and in vivo ischemic stroke model [19]. Furthermore, increased actin depolymerization via the cofilin-related pathway induced β-amyloid aggregation and synaptic loss in a model of TBI [57]. Additionally, we observed significant impairment of cognitive function after ICH in both acute and chronic phases. Our results revealed a noteworthy increase in infarct volume from day 1 to day 3, implying the occurrence of neuronal loss and tissue damage. Subsequently, from day 21 to day 28, there was a shift toward ventricular enlargement, indicating that PSCI may be a consequence of the initial neuronal loss following tissue injury [20]. In line with this evidence, cofilin inhibition in this study rescued synaptic loss represented by increased pre- and post-synaptic protein expression and improved cognitive functions 7 days following the injury.

**Figure 7:**
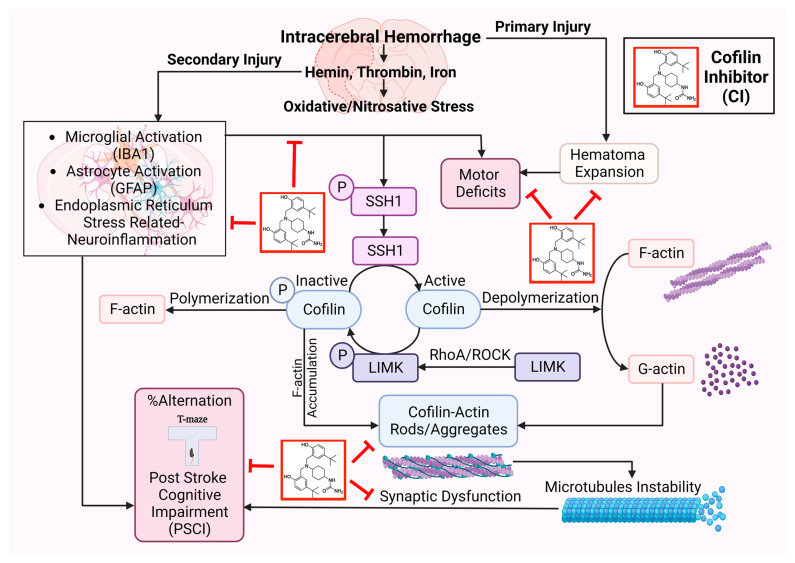
Cofilin signaling, including upstream and downstream pathways affected by secondary injury after ICH. Molecular changes impact motor and post-stroke cognitive impairments (PSCI). Cofilin Inhibitor (CI) reversed these effects following ICH.

In conclusion, CI protected ICH-induced injury by reducing hematoma volume, microglial activation, cofilin rods/aggregates, neuroinflammation, and improved neurobehavioral outcomes following ICH by reducing ER stress-related neuroinflammation subsequently preventing inflammasomes and cell death (Figure 7). CI treatment decreased the brain expression of BIP and CHOP, NLRP3, caspase-1, IL-1β, and caspase-3 and, on the other hand, upregulated PSD-95 and synaptophysin on day 7 after ICH, eventually resulting in significant improvement in motor and PSCI functions. CI treatment also attenuated the detrimental effects of cofilin rods/aggregates, microglia, and astrocytes activation, and oxidative/nitrosative stress by downregulating the expression level of cofilin rods/aggregates, IBA1, GFAP, iNOS, and 3-NT, suggestive of reduced neuroinflammation and that might have led to the recovery of behavioral outcomes on day 7 after ICH. Our findings reveal that CI treatment reduced the pathology associated with neuroinflammation.

The limitations of our study include using young male mice; thus, future studies merit addressing the sex and age impact of CI treatment in the ICH model as well as the outcome of 60 days of survival. In conclusion, the results elicited the molecular mechanisms of CI targeting microglial activation, neuroinflammation, motor impairments, and PSCI. Our finding proposed a novel potential therapeutic alternative targeting neuroinflammation after ICH.

## 4. Materials and Methods

### 4.1. Animals and Experimental Design

All animal studies were approved by the University of Toledo Animal Care and Use Committee and conducted according to the guidelines of the National Institutes of Health. Mice were housed under controlled laboratory conditions, 12 h. light/dark cycle, controlled temperature, and controlled humidity, with access to food and water. This study was carried out using male C57BL/6-mice 8–14 weeks (Jackson Laboratories, Bar Harbor, ME, USA) in strict accordance with the recommendations in the guide for the care and use of laboratory animals from the Department of Laboratory Animal Resources (DLAR), at the University of Toledo, Health Science Campus.

### 4.2. Stereotaxic Injections

Clostridium Collagenase (Sigma-Aldrich, St. Louis, MO, USA) was injected into the left striatum of mice to induce ICH using stereotaxic injection (Stoelting, Wood Dale, IL, USA). Mice were under isoflurane anesthesia, then a small hole in the skull atop the left striatum was made, and a Hamilton syringe needle was injected with the collagenase (0.6 µL, 0.09 units). The stereotaxic coordinates utilized for collagenase infusion were 2 mm lateral to the midline, −1 mm anterior to bregma, and 3 mm depth from the surface. Next, the needle was gradually withdrawn over 10 min, and the wound was closed. After that, the mice were placed on a heated pad for 10 min and transferred to a clean cage. Needle insertion without collagenase injection was performed in sham mice (control group).

### 4.3. Experiment 1

The study evaluated the therapeutic effect of a CI in the ICH mouse model. A total of 50 male C57BL/6-mice 8–14 weeks (Jackson Laboratories) mice were subjected to random assignment, dividing them into three groups: sham/control group (needle insertion only), ICH + vehicle group, and treatment groups [ICH + CI (25 mg/kg), ICH + CI (50 mg/kg), and ICH + CI (100 mg/kg)]. The vehicle that was used to dissolve CI included a proportionate mixture of inert solubilizing agent, DMSO, and Tween 20. Vehicle or different concentrations of CI were administered as a single i.v. injection () 3 h. following ICH and i.p. every 12 h. for 3 days. Animals were trained by an experienced observer blinded to the study design on the rotarod, grip strength, and inverted screen for three days and 24 h before ICH was considered baseline. Rotarod and grip strength were performed from day 1 to day 7 after ICH. Inverted screen and NDS were evaluated on days 1, 3, 5, and 7 following ICH, and T-maze was used to assess the spatial working memory from day 4 to 7. Mice were sacrificed on day 7, brains were harvested for hematoma volume measurement, and blood was collected for cofilin, TNF-α, and IL-1β assessment (experimental design shown in Figure 1A).

### 4.4. Experiment 2

To study the anti-neuroinflammatory properties of CI, a total of 12 male C57BL/6-mice (8–14 weeks, Jackson Laboratories) were randomly divided into three groups: sham (control group), ICH + vehicle group, and ICH + CI (50 mg/kg). Mice were euthanized on day 7, and brains were extracted for WB.

### 4.5. Neurobehavioral Tests

A. Neurological deficit scoring (NDS)

NDS is a scoring system consisting of 28 points, which is used to evaluate sensory and motor impairments following ICH [58]. After ICH, seven tests, namely body symmetry, gait, climbing, circling behavior, whisker response, compulsory circling, and front limb symmetry, were assessed and graded on a scale from zero (indicating no deficit) to four (indicating severe deficit).

B. Rotarod test

Motor coordination deficits after ICH were assessed using the rotarod test, following a previously described protocol [29]. In brief, the rotarod apparatus consisted of a rotating rod set initially at 1 RPM, with speed increasing by 1 RPM every 10 s until the mouse fell off. The trial was completed when the mouse lost its balance and fell from the rod, and the time was recorded. To establish the baseline performance, animals were trained on the rotarod 3–7 days and 24 h. before the surgery (each animal was subjected to the test three times per trial, and the average time to fall value was calculated and utilized for subsequent statistical analysis).

C. Grip strength test

Grip strength measurements were conducted to assess the strength of the forelimb muscles in mice following ICH [29]. The forelimbs of the mice were positioned on a pull bar assembly while the experimenter held the mouse by its tail. The maximum force exerted by the mouse, as displayed on the digital screen, was recorded (each animal underwent the test three times per trial, and the average value was employed for statistical analysis). The scores recorded 3–7 days before the ICH procedure were considered baseline measurements.

D. Inverted screen test

The inverted screen test assesses motor strength/coordination using all four limbs [59]. Untrained mice are placed individually on top wire mesh in the center and then rotated 180°. The time when the mice fall off is recorded, or it can be removed after 60 s. The inverted screen test was performed 3–7 days before ICH (baseline reading). Three test measurements were recorded, and the average value was employed for each time point and considered for statistical analysis.

E. T-maze test

Spatial learning and cognitive impairment were assessed using T-maze after ICH. The spontaneous alternation protocol was employed as described previously [20,60] with minor modifications. During each trial, the mouse was initially placed on the central arm of the maze and allowed to explore both arms for 2 min. Once the mouse selected a specific goal arm, the arm was blocked, and after 30 s, the mouse was removed from the maze. A brief inter-trial interval followed, during which the mouse was placed back into the cage for 30 s while the block was removed from the maze. Subsequently, the mouse was placed in the start arm of the T-maze, enabling the mouse to choose between the available arms. The experiment consisted of two sets per day, conducted over four days. The correct choice was recorded when the mouse selected a different arm during the second run compared to its choice in the first run within a particular set. Conversely, an incorrect choice was recorded when the mouse selected the same arm as in the previous run. The percentage of alteration, which reflects the rate of spontaneous alternation, and the rate of side preference were calculated based on the recorded choices, providing insights into spatial learning and behavioral patterns in the T-maze paradigm.

%Alteration = (Correct choice/Total set) × 100

%Side Preference = (Preferred side/Total run) × 100

### 4.6. Western Blotting (WB)

On day 7, the mice were euthanized with CO_2_, and brains were collected for WB analysis. Brain tissues were collected from the hemorrhagic margins (ipsilateral) using a 1 mm diameter micro-punch needle. Brain tissues were mechanically disrupted in a cold RIPA buffer (Thermofisher Scientific, Waltham, MA, USA) and a protease inhibitor cocktail (Sigma-Aldrich, St. Louis, MO, USA) to complete homogenization. Protein concentrations were quantified and determined using Bradford reagent (Bio-Rad, Hercules, CA, USA). Protein samples were then loaded onto 10% or 12% SDS-polyacrylamide gels in equal amounts. Protein samples were separated utilizing gel electrophoresis, transferred onto a polyvinylidene fluoride membrane (PVDF), and blocked with 5% milk or 5% BSA for 1 h. Primary antibodies were added as follows, and the membranes were incubated overnight at 4 °C: rabbit anti-SSH1 (1:1000; Abcam, Waltham, MA, USA), rabbit anti-cortactin (1:1000; Cell Signaling, Technology, Inc. Denver, MA, USA), rabbit anti-P-LIMK (1:1000; Cell Signaling), rabbit anti-LIMK (1:1000; Cell Signaling), rabbit anti-p-cofilin (1:500; cell signaling), rabbit anti-cofilin (1:1000; Cell Signaling), rabbit anti-BIP (1:1000, Cell Signaling), rabbit anti-calreticulin (1:1000, Cell Signaling), rabbit anti-EIF-2α (1:1000, cell signaling), rabbit anti-p-EIF-2α (1:1000, Cell Signaling), mouse anti-CHOP (1:1000, Cell Signaling), rabbit anti-NLP3 (1:500, Cell Signaling), mouse anti-IL-1β (1:1000, Cell Signaling), rabbit anti-IL-18 (1:1000, Cell Signaling), rabbit anti-caspase-1 (1:1000, Cell Signaling), rabbit anti-caspase-3 (1:1000, Cell Signaling), mouse anti-synaptophysin (1:1000; Abcam), mouse anti-PSD95 (1:1000, Cell Signaling), rabbit anti-GFAP (1:1000; Abcam), rabbit anti-GLT-1 (1:1000, Cell Signaling), and rabbit anti-BDNF (1:1000, Abcam). After 24 h, the blots were washed three times with TBST on a shaker and incubated with secondary antibodies (horseradish peroxidase (HRP)-conjugated goat anti-mouse, or goat anti-rabbit, Cell Signaling) for 1 h at room temperature on a rocker. The images were acquired using a Syngene Imaging System (Frederick, MD, USA). The images were assessed using ImageJ Software 1.53t (National Institute of Health, Bethesda, MD, USA). β-actin (1:1000, Cell Signaling) was used as a loading control.

### 4.7. Histology and Hematoma Volume Measurement

For these experiments, different cohorts of mice were anesthetized on day 7 with Ketamine (100 mg/kg)/Xylazine (10 mg/kg) (i.p.) and then transcardially perfused with cold 1× phosphate-buffered saline (PBS) followed by cold 4% paraformaldehyde. Mice brains were harvested and placed in cold 4% paraformaldehyde for 24 h. Brains were embedded in paraffin and sections (5 mm) using a rotary microtome. Brain sections were deparaffinized and hydrated in graded methanol, followed by Luxol fast blue/Cresyl violet staining to hematoma volume. Hematoma volume was measured by using the method published previously [29]. Additional sections were made for immunohistochemistry.

### 4.8. Immunohistochemistry

Deparaffinized mice brain sections, followed by antigen retrieval process for 15 min in a pressure cooker, washed three times with 1× PBS for a duration of 5 min and blocked with 5% BSA dissolved in TBST for another 2 h. on a rocker. After that, the primary antibodies were added and incubated overnight at 4 °C, 1:1000; mouse anti-cofilin (Abcam), 1:1000; rabbit anti-IBA1 (Wako, Japan), rabbit anti-3NT (1:200, Cell Signaling), mouse anti-iNOS (1:200, Sigma). After 24 h, the slides were washed three times with 1× PBS, and the secondary antibodies were added and incubated in the dark container for 1 h. at room temperature Alexa Fluor donkey anti-mouse IgG (1:1000; Jackson, Immunoresearch, West Grove, PA, USA), (Taxes red labeled donkey anti-rabbit IgG (1:1000; Jackson, Immunoresearch). The slides underwent a series of 3 washes using 1× PBS and were mounted with DAPI (Molecular Probes, Eugene, OR, USA). The images of the localization of cofilin in microglia and iNOS with 3NT were acquired using fluorescent microscopy. Cofilin fluorescence intensity, rod fluorescence intensity, and rod length around the hemorrhagic region were measured using ImageJ software 1.53t (NIH), and the number of microglia was determined by counting the activated cells around the hemorrhage using ImageJ software 1.53t (NIH).

### 4.9. Microglia Morphology Quantification

Morphological alterations of microglia using mouse brain sections were analyzed using a method published earlier [20]. In brief, single-cell microglia were transformed into photomicrographs of binary and outline images. A box-counting dimension method was applied to interpret the morphological features of microglia using ImageJ with the plugins of FracLac for ImageJ analysis [61].

### 4.10. ELISA

The level of cofilin in plasma was measured by following the manufacturer’s instructions for a commercially available mouse cofilin enzyme-linked immunosorbent assay (ELISA) (Bio-Techne, Minneapolis, MN, USA). The levels of TNFα and IL-β in serum were assessed using the manufacturer’s recommendations (R&D, Minneapolis, MN, USA).

### 4.11. Statistics and Data Analysis

Data were normally distributed, confirmed by Shapiro–Wilk’s test. The experimental findings were expressed as mean ± SEM, and statistical significance was determined at *p* < 0.05. Data were analyzed by one-way or two-way ANOVA, followed by Sidak *post hoc* comparisons. Data with equal variances and normal distribution were evaluated using repeated-measures ANOVA followed by Sidak *post hoc* comparisons. A two-tailed unpaired Student’s *t*-test was employed to assess the mean of the two groups. All statistics analyses were completed using GraphPad Prism version 10.00 (GraphPad Software, San Diego, CA, USA).

## Data Availability

Data is contained within the article and Supplementary Materials.

## Supplementary Materials

The following supporting information can be downloaded at https://www.mdpi.com/article/10.3390/ph17010114/s1. Figure S1: CI improved motor deficits and inflammatory markers; Figure S2: Mortality rates after treatment with different concentrations of CI; Figure S3: CI reduced the levels of 3-NT with iNOS; Figure S4: Original WB for Figure 3; Figure S5: Original WB for Figure 4; Figure S6: Original WB for Figure 5; Figure S7: Original WB for Figure 6.
