# Supplementary material for: Cofilin Inhibitor Improves Neurological and Cognitive Functions after Intracerebral Hemorrhage by Suppressing Endoplasmic Reticulum Stress Related-Neuroinflammation
Source: Pharmaceuticals (Basel). 2024 Jan 15;17(1):114. doi: 10.3390/ph17010114 (PMC10818666; doi:10.3390/ph17010114)
Supplement: Supplementary file 1 [file pharmaceuticals-17-00114-s001.zip › pharmaceuticals-2769398-supplementary.pdf]

Supplementary Figures

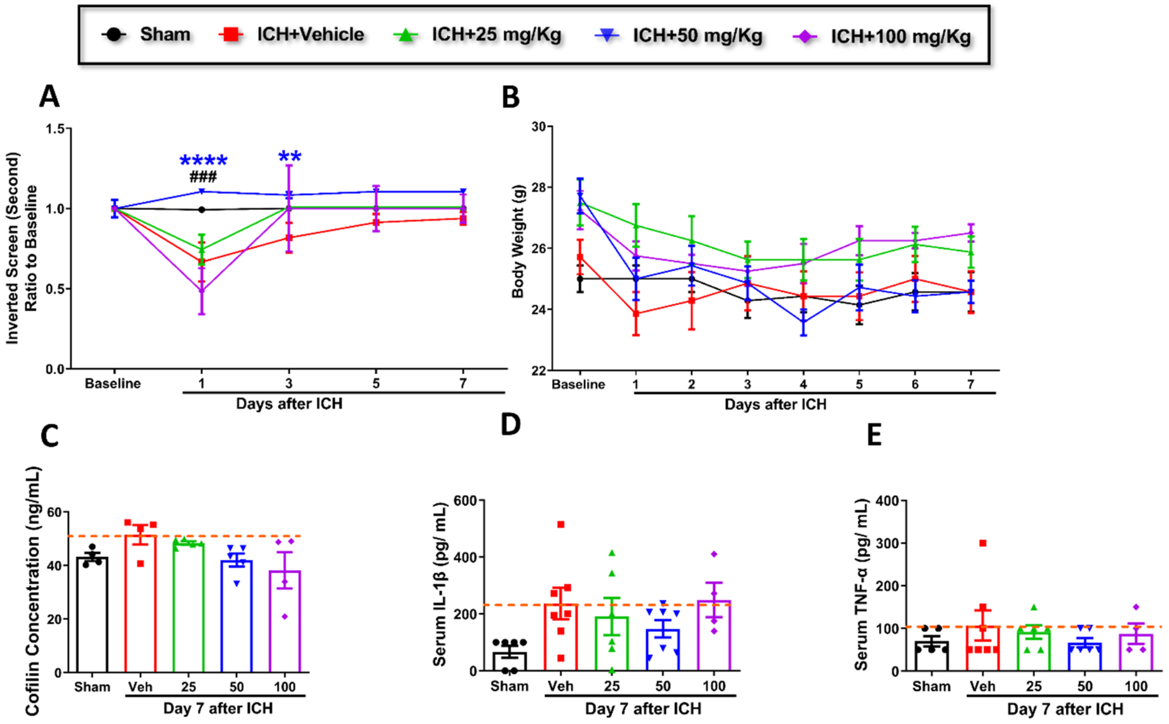

Figure S1. CI improved motor deficits and inflammatory markers. Effect of different doses of cofilin inhibitor (CI, 25, 50, and 100 mg/kg) on inverted screen, body weight, and blood profile ICH in mice. (A) Inverted screen latency time was recorded on days 1, 3, 5, and 7 after ICH and showed significant improvement on days 1 and 3 with CI 50 mg/kg compared to the vehicle after ICH. (B) No changes were observed in body weight among different groups. (C) plasma level of cofilin, (D) serum level of IL-1β and (E) serum level of TNF-α were decreased in the CI-treated group compared with the vehicle group after ICH, but the data was not significant. (n=6-7 per group, Two-way ANOVA or one-way ANOVA followed by Sidak's *post hoc* comparisons). Data are indicated as mean ± SEM, where $p < 0.05$ was considered significant. *Difference of ICH + CI group relative to ICH + vehicle group (ns = not significant, ** $p < 0.01$, **** $p < 0.0001$) and #Difference of ICH + vehicle compared to the sham group ### $p < 0.001$).

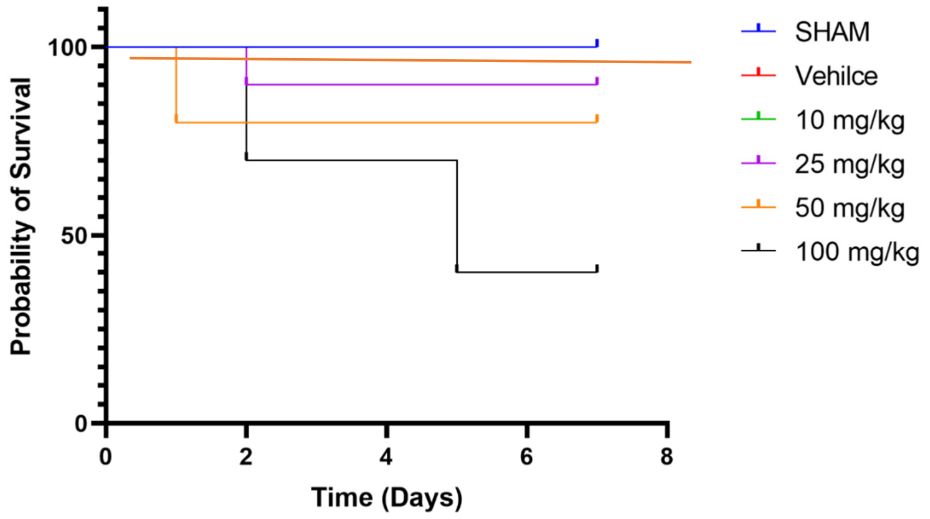

Figure S2: Mortality rates after treatment with different concentrations of CI. 60% mortality was observed in 100mg/kg; 20% mortality in 50mg/kg, 10% in 10mg/kg and no mortality in vehicle. Kaplan-Meier survival curve of mice. Differences in survival between different groups were analyzed by log rank test ($p < 0.05$).

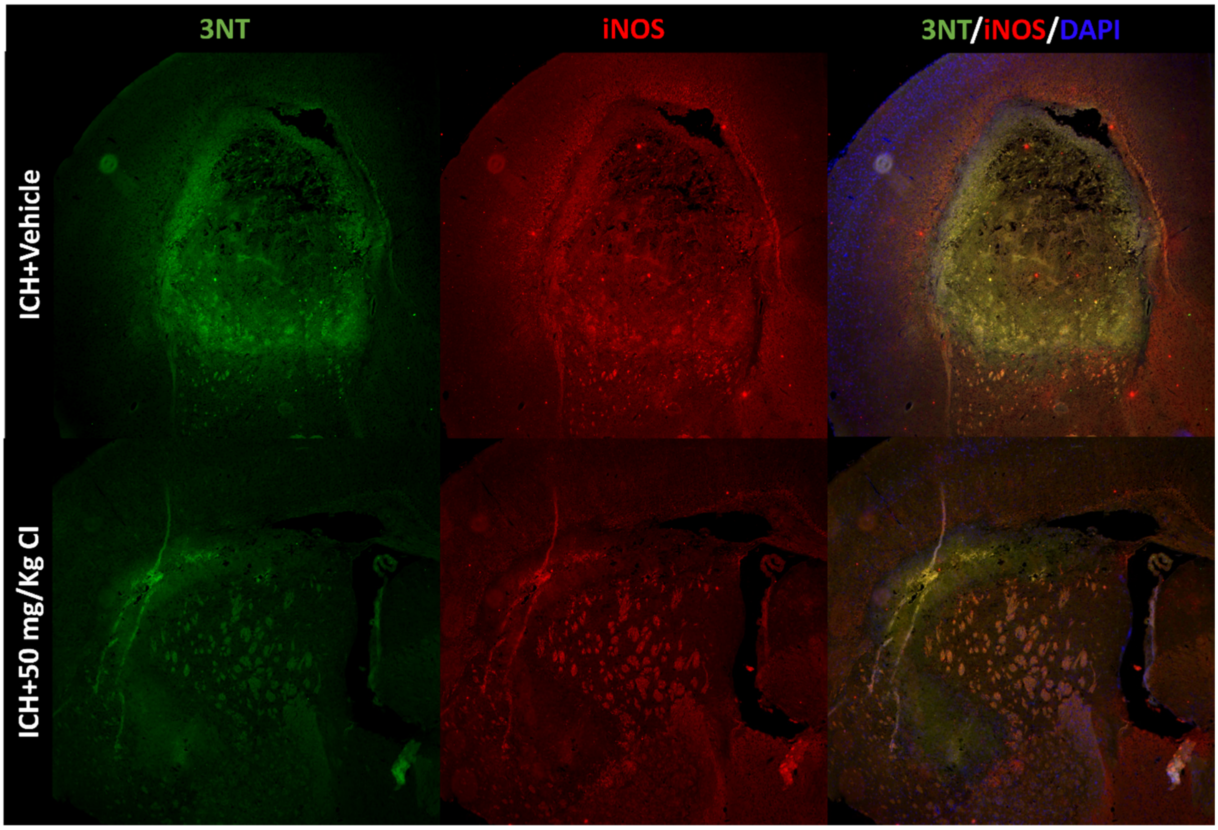

Figure S3. CI reduced the levels of 3-NT with iNOS. The effect of CI (50 mg/kg) on oxidative/nitrosative stress after ICH. Immunohistochemical staining showed that the colocalization of 3-NT with iNOS was diminished in the CI-treated group relative to the vehicle group after ICH on day 7.

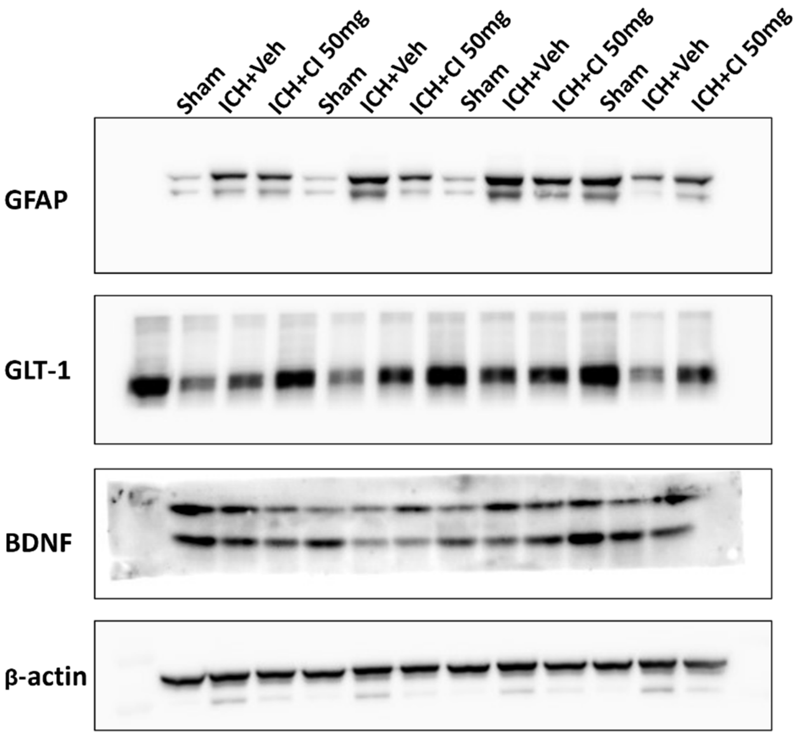

Figure S4: Original Western blots for Figure 3.

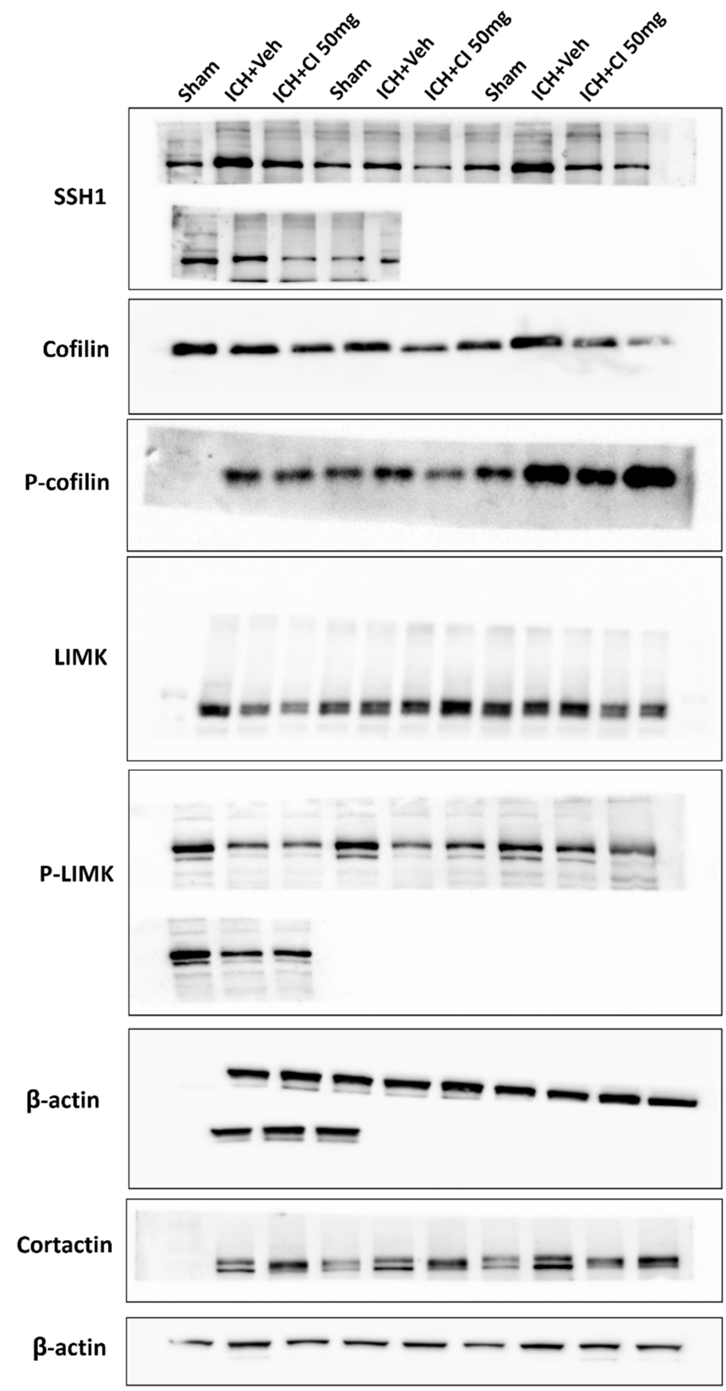

Figure S5: Original Western blots for Figure 4.

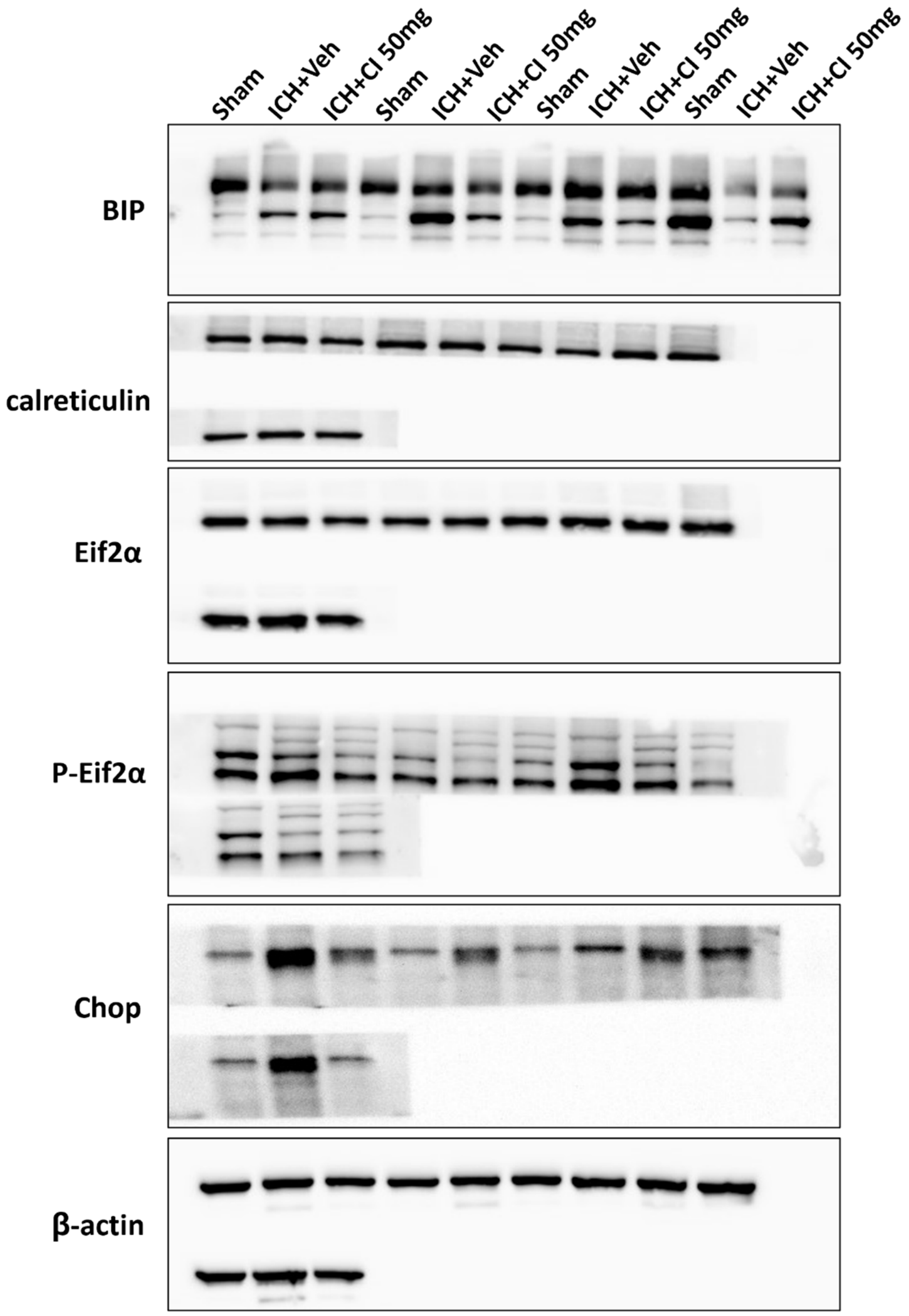
Sham
ICH+Veh
ICH+CI 50mg
Sham
ICH+Veh
ICH+CI 50mg
Sham
ICH+Veh
ICH+CI 50mg
Sham
ICH+Veh
ICH+CI 50mg
BIP
calreticulin
Eif2α
P-Eif2α
Chop
β-actin

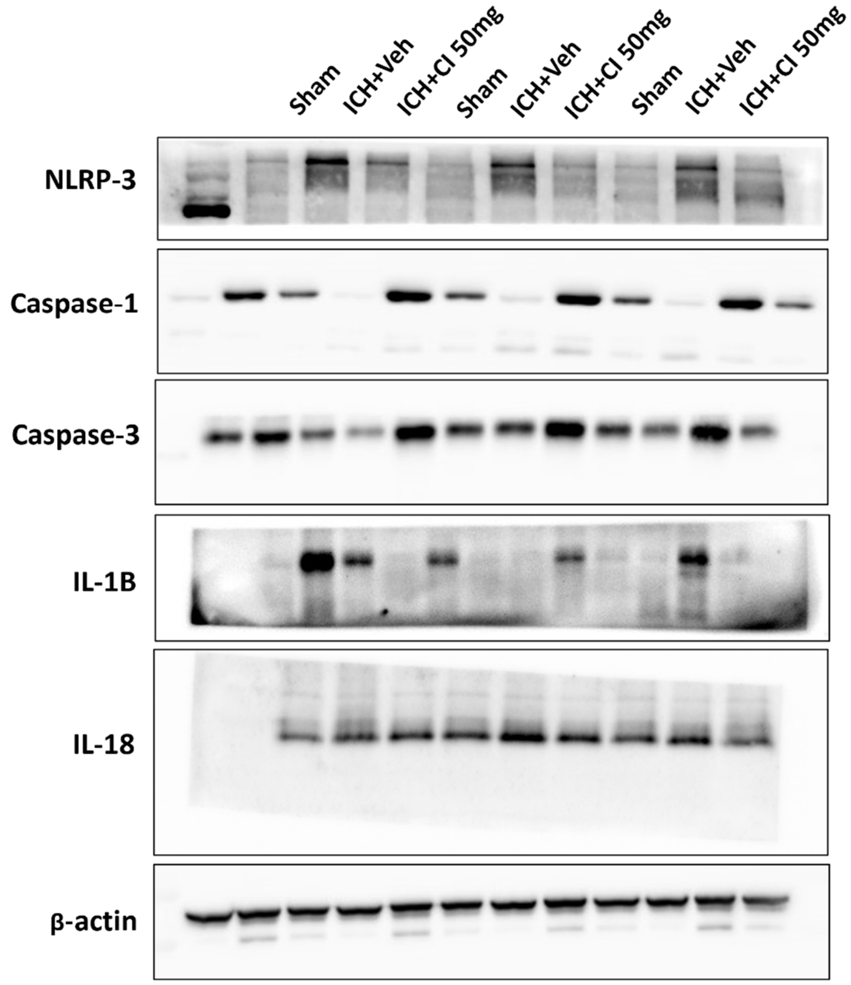

Figure S6: Original Western blots for Figure 5.

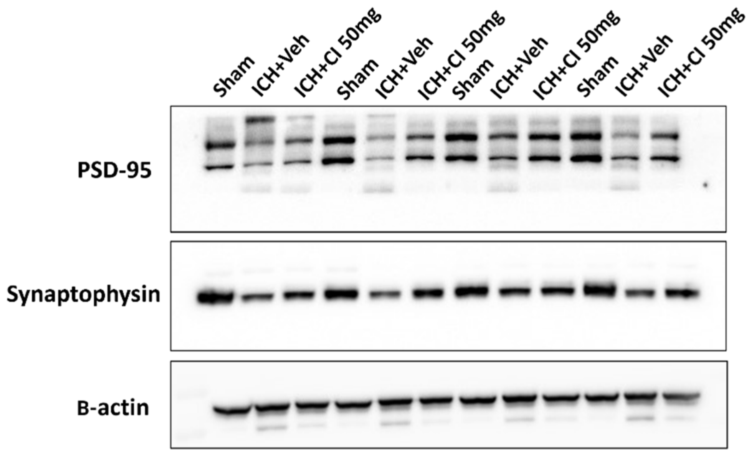

Figure S7: Original Western blots for Figure 6.
